# IgG4-related aortitis/periaortitis and periarteritis: a distinct spectrum of IgG4-related disease

**DOI:** 10.1186/s13075-020-02197-w

**Published:** 2020-05-04

**Authors:** Linyi Peng, Panpan Zhang, Jieqiong Li, Zheng Liu, Hui Lu, Liang Zhu, Xiaorong Wang, Fei Teng, Xuemei Li, Huifang Guo, Yunyun Fei, Wen Zhang, Yan Zhao, Xiaofeng Zeng, Fengchun Zhang

**Affiliations:** 1 Department of Rheumatology, Peking Union Medical College Hospital, Chinese Academy of Medical Science & Peking Union Medical College, Key Laboratory of Rheumatology and Clinical Immunology, Ministry of Education & National Clinical Research Center for Dermatologic and Immunologic Diseases (NCRC-DID), Beijing, China; 2 Department of Radiology, Peking Union Medical College Hospital, Chinese Academy of Medical Science & Peking Union Medical College, Beijing, China; 3 Department of Rheumatology, The First People’s Hospital of Yangquan, Yangquan, China; 4 Department of Nephrology, Peking Union Medical College Hospital, Chinese Academy of Medical Science & Peking Union Medical College, Beijing, China; 5 Department of Rheumatology, The Second Hospital of Hebei Medical University, Shijiazhuang, China

**Keywords:** IgG4-related disease, Aortitis, Periaortitis, Periarteritis, Retroperitoneal fibrosis

## Abstract

**Background:**

Large vessels could be involved in immunoglobulin (Ig)-G4-related disease (IgG4-RD). This study aimed to clarify the clinical features and evaluate the treatment efficacy for IgG4-RD with aortitis/periaortitis and periarteritis (PAO/PA).

**Methods:**

This study prospectively enrolled 587 patients with IgG4-RD with a follow-up time of more than 6 months. The distribution of IgG4-related PAO/PA was classified into four types: type 1, thoracic aorta; type 2a, abdominal aorta; type 2b, abdominal aorta and iliac artery; type 2c, iliac artery; type 3, thoracic and abdominal aorta; and type 4, other arteries. Patient’s demographic data, clinical characteristics, laboratory parameters, and treatment efficacy were analyzed.

**Results:**

Of 587 IgG4-RD patients, 89 (15.2%) had PAO/PA. The average age was 58.3 ± 11.1 years, with male predominance (85.4%). Vessels affected were as follows: abdominal aorta (83.1%), iliac artery (70.8%), thoracic aorta (13.5%), and other vessels (13.5%). The most prevalent distribution type of IgG4-related PAO/PA was type 2b, with 74 (83.1%) patients, followed by type 2a, type 2c, type 3, and type 1. Fifty-five (61.8%) PAO/PA patients had hydronephrosis, with renal insufficiency occurring in 43 (48.3%), and 31 (34.8%) PAO/PA patients had D-J stent drainage due to severe ureteral obstruction. After treatment with a glucocorticoid and immunosuppressants, 82% patients achieved remission with shrinking of the perivascular mass by more than 30%.

**Conclusions:**

IgG4-RD with PAO/PA was distinct from non-PAO/PA in demographic features, organ involvement distribution, inflammatory markers, and serum IgG4 and IgE. The most common affected vessel was the abdominal aorta, and most patients responded well with treatment.

## Background

Immunoglobulin (Ig)-G4-related disease (IgG4-RD) is a fibro-inflammatory condition characterized by tumor-like swelling of affected organs, with elevated serum IgG4 and massive infiltration of lymphocytes and plasma cells in involved organs [1–5]. It is a highly heterogeneous disease entity that could affect nearly any organ and often present with multiorgan involvement [6], and the most affected tissues are the lacrimal gland, submandibular gland, lymph node, and pancreas [6–8]. Evidence suggested that IgG4-RD could affect various organs including the vascular system as aortitis/periaortitis/periarteritis (PAO/PA) [9–12]; additionally, the most frequently involved blood vessel is the aorta, and a medium-sized artery, such as the iliac artery and carotid artery, may be a potential target [5].

PAO/PA was reported to be present in 10–30% of overall IgG4-RD, and they may appear as an isolated lesion of IgG4-RD. Studies had investigated the histopathological diagnosis of the vascular involvement of IgG4-RD, vessel distributions, concomitant non-vascular lesions, treatment efficacy, or potential etiology [5, 10, 13, 14]. However, the lack of large prospective studies may hinder our understanding of the complete clinical picture of IgG4-related PAO/PA because of its heterogeneity. Moreover, early diagnosis and treatment are essential to minimizing irreversible organ damage or unnecessary surgical intervention [6], such as aneurysmal dilation or rupture of the aorta, or irreversible renal dysfunction due to the obstruction of ureters and hydronephrosis. Therefore, to further understand and achieve better management of IgG4-related PAO/PA, we aimed to analyze the clinical patterns of IgG4-related PAO/PA, distribution of affected vessels, and treatment efficacy.

## Methods

### Inclusion and exclusion criteria

In our prospective cohort of IgG4-RD carried out in the Peking Union Medical College Hospital (registered as ClinicalTrials.gov ID: NCT01670695), 587 patients were enrolled from January 2011 to September 2018, fulfilling the 2011 comprehensive diagnostic criteria [15, 16]. Each IgG4-RD patient had follow-up time of more than 6 months. The diagnosis of IgG4-RD was based on the following criteria: (1) a clinical examination showing characteristic diffuse/localized swelling or masses in single or multiple organs, (2) an elevated serum IgG4 concentration (> 135 mg/dL), and (3) a histopathologic examination showing (a) marked lymphocytic and plasma cell infiltration and fibrosis or (b) infiltration of IgG4+ plasma cells (a ratio of IgG4+/IgG+ cells > 40% and > 10 IgG4+ plasma cells per high power field). Patients with other autoimmune diseases, active/severe infection, and malignant disease were excluded. Patients combined with antineutrophil cytoplasmic antibody-associated vasculitis were excluded. Patients’ affected organs and evaluation of treatment efficacy were determined by clinical symptoms, physical examinations, histological pathology, and imaging, including ultrasonography, computed tomography (CT), magnetic resonance imaging (MRI), or positron emission tomography/computed tomography (PET/CT). Allergy history information was collected using the criteria from the European Academy of Allergy and Clinical Immunology. The study was conducted in compliance with the Declaration of Helsinki and was approved by the Ethics Committee of Peking Union Medical College Hospital (number S-442). All patients signed written informed consent.

### Detection of PAO/PA by CT, MRI or PET-CT

A patient was diagnosed with PAO/PA if one or more of the following conditions are present: (1) vessel wall thickening, (2) vessel wall enhancement on contrast imaging, (3) soft tissues around blood vessels with circumferential enhancement on CT or MRI, (4) and ^18^fludeoxyglucose avidity within a vessel wall or perivascular region on PET/CT [5, 9]. Improvement of PAO/PA was defined as shrinking of perivascular soft tissues and reduced vessel wall thickness by averaging the 2 dimensions of greatest change on CT. Luminal dilatation at the time of periaortic/periarterial lesion diagnosis was defined as aneurysm, in which the luminal diameter was > 1.5 times wider than normal (> 45 mm at the thoracic aorta and > 30 mm at the infra-renal abdominal aorta) [17, 18]. This study included 89 PAO/PA patients; 8 patients with retroperitoneum lesion but without vessels affected were excluded, and patients with malignant tumor during follow-up were excluded. The treatment efficacy was evaluated by clinical characteristics, laboratory parameters, and CT scans.

For 43 patients with IgG4-related PAO/PA with renal impairment, the estimated glomerular filtration rate (eGFR) was calculated before and after 3 months of treatment using the Chronic Kidney Disease-Epidemiology Collaboration formula. Removal time of the ureteral stent was decided by consensus among rheumatologists and urologists after determining whether the soft tissue around the ureter was reduced, ureteral compression was relieved, and hydronephrosis and renal function improved [19]. We monitored renal function and ultrasonography of the kidney every 2 weeks during the first 2 months after stent removal. If renal function was stable and hydronephrosis was not aggravated, stent removal was considered successful.

**Fig. 1:**
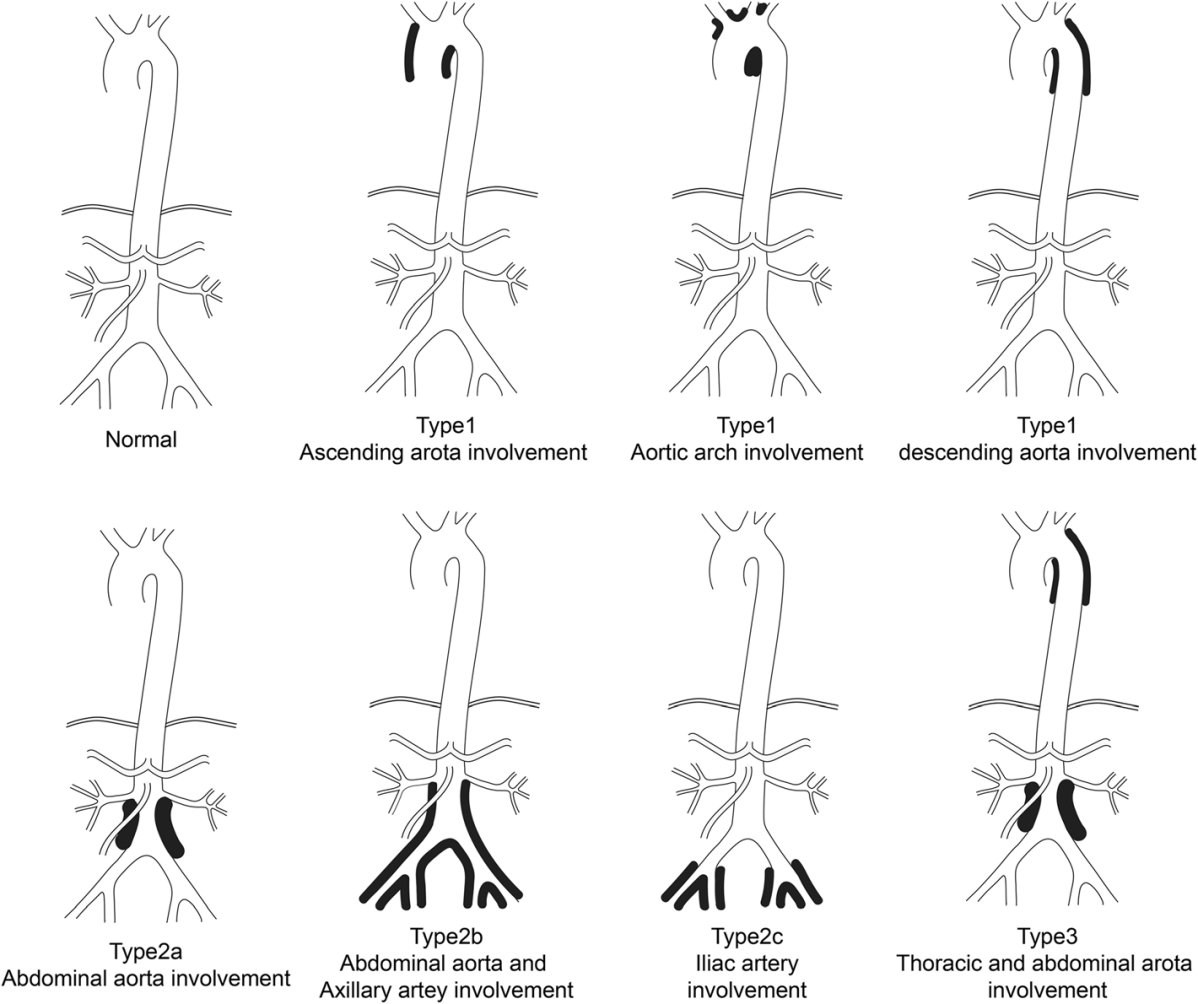
Classification of immunoglobulin G4-related disease aortitis/periaortitis and periarteritis distribution

Based on Ozawa et al.’s study of vessel distribution [5] and our clinical experience, PAO/PA in our study was classified into four types according to the distribution of affected vessels: type 1, thoracic aorta; type 2, abdominal aorta and iliac artery involved, including type 2a, abdominal aorta, type 2b, abdominal aorta and iliac artery, type 2c, iliac artery; type 3, thoracic and abdominal aorta; and type 4, other arteries (Fig. 1).

### Clinical data and laboratory parameters

Patients’ data including age, sex, disease duration, history of allergy, treatment strategy, symptom onset, organs affected, and follow-up time were collected. Disease duration refers to the interval between symptom onset and diagnosis. The IgG4-RD responder index (RI) (2015 version) at baseline and each follow-up was evaluated [20]. Laboratory parameters included routine blood analysis; liver function; kidney function; serum IgG, A, and M; serum IgG subclass; total serum IgE; rheumatoid factor, C3 and C4, erythrocyte sedimentation rate (ESR), and hypersensitive C-reactive protein (hsCRP) tests.

### Flow cytometry of CD19^+^CD24^−^CD38^hi^ plasmablast/plasma cells

Peripheral blood mononuclear cells from IgG4-RD patients were separated by Ficoll gradient centrifugation. B cell subpopulations were stained with PE-Cy7-conjugated anti-CD19, FITC-conjugated anti-CD24, and APC-conjugated anti-CD38 (BD Bioscience, USA). Plasmablast/plasma cell was defined as CD19^+^CD24^−^CD38^hi^. Altogether, 93 patients tested CD19^+^CD24^−^ CD38^hi^ plasmablast/plasma cells at baseline, including 18 IgG4-RD PAO/PA patients and 75 non-PAO/PA patients.

### Treatment efficacy assessment

According to the comparison CT scans before treatment and at 3 months after treatment, the reduction of soft tissue around affected vessels was categorized into three types: 0–30%, 31–70%, and > 70%.

Treatment response was assessed by evaluating the changes in IgG4-RD RI scores and was divided into complete response (CR), partial response (PR), and no effect (NE, including no improvement or exacerbation). IgG4-RD RI scores < 3 and decline of ≥ 2 were recognized as CR; IgG4-RD RI score decline of ≥ 2 but remained ≥ 3 were recognized as PR. If patients’ IgG4-RD RI score was 3 points at the beginning, PR was considered a 1-point decrease after the therapy. Patients lacking apparent changes in mass sizes and/or clinical manifestations and IgG4-RD RI score decline of < 2 were considered NE [21]. Clinical relapse was defined as reappearance of clinical symptoms or imaging findings were worsened with or without elevated serum IgG4 levels [21, 22].

### Statistical methods

Statistical analyses were performed using the IBM SPSS Statistics version 24.0 software (IBM, Armonk, NY, USA), Adobe Illustrator CC 2014 (Adobe Systems, San Jose, CA, USA), and the Prism software version 6.1 (GraphPad Software, La Jolla, CA, USA). Data were reported as means ± standard deviation or median (quartile 1 [Q1]–quartile 3 [Q3]). Normally distributed data between two groups were analyzed using independent-samples *t* tests or paired-samples *t* tests, and a one-way analysis of variance was used to compare groups. Categorical data were analyzed using the chi-square test or Fisher’s exact tests, while non-normally distributed data were analyzed using the rank sum test. A two-tailed *P* value < 0.05 was considered statistically significant.

## Results

### Demographic characteristics of IgG4-RD with PAO/PA

Among 587 IgG4-RD patients, 89 patients (76 men and 13 women) were included in the IgG4-related PAO/PA group and 498 patients were included in the non-PAO/PA group. Of the IgG4-RD patients with PAO/PA, 24 (27.0%) had PAO/PA alone, while the other patients (*n* = 65, 73.0%) had multiple organ involvement. Demographic features of IgG4-RD with/without PAO/PA are shown in Table 1. The age of PAO/PA patients was 58.3 ± 11.1 years, with a male/female ratio of 5.85/1. The IgG4-RD RI was 10.8 ± 5.3 at baseline. Male patients (59.5 ± 9.7 years) were older than female patients (51.3 ± 16.1 years) at disease onset (*P* = 0.015). Moreover, 25 (28.1%) PAO/PA patients had an allergic history. The median follow-up time was 30 (15, 52) months.

**Table 1:** Demographic features of IgG4-RD with/without PAO/PA

Demographic features	PAO/PA (***n*** = 89)	Non-PAO/PA (***n*** = 498)	***P*** value
Age (years)	58.3 ± 11.1	52.6 ± 13.8	< 0.001*
Male/female ratio	5.85/1	1.35/1	< 0.001*
Disease duration (month), M (Q1–Q3)	6 (2.5–36)	12 (6–36)	< 0.001*
History of allergy (*n*, %)	25 (28.1)	267 (53.6)	< 0.001*
IgG4-RD RI	10.8 ± 5.3	9.8 ± 5.2	0.103
Number of organs involved	2.9 ± 1.9	3.0 ± 1.7	0.126
Patients with single organ involved (*n*, %)	24 (27.0)	66 (13.3)	0.001*

*Statistical significance

We compared the prevalence of allergic diseases in IgG4-PAO/PO patients with/without extra-glandular involvement. The results showed that allergic diseases were more common in patients with lacrimal and/or salivary gland lesions than in those without (53.6% versus 16.4%, *P* < 0.001).

Of 89 patients with PAO/PA, 35 (39.3%) patients were diagnosed as having definite IgG4-RD, 1 (1.1%) was probable, and 53 (59.6%) patients were possible. Nine (10.1%) patients underwent perivascular mass biopsy, while the rest of the patients underwent biopsy of other involved organs, including the submandibular gland, lacrimal gland, kidney, lymph node, and lung.

### Symptoms at disease onset in IgG4-RD patients with PAO/PA

Symptoms at disease onset in IgG4-RD patients with PAO/PA are shown in Table 2. Pain was the most prevalent symptom observed; among all patients, 57 (64.0%) patients had onset symptoms of back pain (32, 36.0%) and abdominal pain (25, 28.1%). Fourteen patients (15.7%) had lower limb edema. Other onset symptoms included lymph node swelling (22, 24.7%), submandibular gland enlargement (18, 20.2%), cough (11, 12.4%), lacrimal gland enlargement (10, 11.2%), jaundice (8, 9.0%), parotid gland enlargement (5, 5.6%), and nasal congestion (4, 4.5%).

**Table 2:** Onset symptoms and organs involvement of IgG4-RD patients with/without PAO/PA

Symptoms and organs affected at baseline	PAO/PA	Non-PAO/PA	***P*** value
Symptoms at disease onset (*n*, %)			
Back pain	32 (36)	21 (4.2)	< 0.001*
Lymph node swelling	22 (24.7)	137 (27.5)	0.575
Abdominal pain	25 (28.1)	79 (15.9)	0.005*
Submandibular gland enlargement	18 (20.2)	213 (42.8)	< 0.001*
Lacrimal gland enlargement	10 (11.2)	227 (45.6)	< 0.001*
Lower limb edema	14 (15.7)	8 (1.6)	< 0.001*
Cough	11 (12.4)	53 (10.6)	0.632
Nausea and vomiting	12 (11.1)	42 (8.4)	0.129
Jaundice	8 (9.0)	72 (14.5)	0.166
Parotid gland enlargement	5 (5.6)	66 (13.3)	0.042*
Nasal congestion	4 (4.5)	106 (21.3)	< 0.001*
Itching	5 (5.6)	45 (9.0)	0.287
Organs affected (*n*, %)			
Lymph node	33 (37.1)	219 (44)	0.674
Submandibular gland	24 (27.0)	259 (52)	< 0.001*
Pancreas	26 (29.2)	175 (35.5)	0.278
Lung	15 (16.9)	117 (23.5)	0.167
Lacrimal gland	11 (12.4)	266 (53.4)	< 0.001*
Parotid gland	9 (10.1)	90 (18.1)	0.065
Bile duct	11 (12.4)	99 (19.9)	0.094
Paranasal sinus	8 (9.0)	155 (31.1)	< 0.001*
Prostate	12 (15.8)	39 (13.6)	0.632
Kidney	6 (6.7)	44 (8.8)	0.515
Thyroid	2 (2.2)	21 (4.2)	0.378
Pituitary	2 (2.2)	9 (1.8)	0.778
Skin	1 (1.1)	28 (5.6)	0.071

### Laboratory parameters of IgG4-RD patients with PAO/PA

Among IgG4-RD patients with PAO/PA, the serum creatinine level increased in 43 (48.3%) patients (131 [117, 179] μmol/L) (Table 3), including 39 (51.3%) male patients and 4 (30.8%) female patients. ESR and hsCR*P* values were 44 (18–75) mm/h and 6.72 (2.14–24.65) mg/L, respectively. Serum IgG, IgG4, and T-IgE levels were 19.88 ± 8.20 g/L, 4240 (2015, 7730) mg/L, and 170 (95.3, 463.5) KU/L, respectively.

**Table 3:** Laboratory parameters of IgG4-RD patients with/without PAO/PA

Parameters	PAO/PA (***n*** = 89)	Non-PAO/PA (***n*** = 498)	***P*** value
HgB (g/L)	127 ± 21	135 ± 19	< 0.001*
WBC (10^9^/L)	7.9 ± 2.8	7.15 ± 2.55	0.014*
PLT (10^9^/L)	240 ± 87	238 ± 89	0.862
Eos% elevation (%)	22.5	32.4	0.077
ESR (mm/h), M (Q1–Q3)	44 (18–75)	16 (7–40)	< 0.0001*
Elevation of ESR (*n*, %)	61 (77.1%, 61/70)	205 (42.0%, 205/488)	< 0.001*
hsCRP (mg/L), M (Q1–Q3)	6.72 (2.14–24.65)	1.78 (0.72–5.12)	< 0.0001*
Elevation of hsCRP (*n*, %)	56 (70%, 56/80)	150 (37.5%, 150/400)	< 0.001*
IgG (g/L)	19.88 ± 8.20	21.28 ± 14.46	0.292
IgA (g/L)	2.53 ± 1.13	2.16 ± 1.29	0.02*
IgM (g/L), M (Q1–Q3)	0.88 (0.56–1.14)	0.77 (0.54–1.19)	0.573
IgG1 (mg/L), M (Q1–Q3)	9355 (7928–11,325)	8665 (7013–10,600)	0.03
IgG2 (mg/L), M (Q1–Q3)	5705 (4255–7350)	5595 (4290–7520)	0.873
IgG3 (mg/L), M (Q1–Q3)	461 (221–923)	439 (253–841)	0.802
IgG4 (mg/L), M (Q1–Q3)	4240 (2015–7730)	8310 (3250–17,075)	< 0.0001*
T-IgE (KU/L), M (Q1–Q3)	170 (95.3–463.5)	332 (119–720.5)	0.025*
Cr (μmol/L)	98.5 (73–131.3)	67 (57.7–78)	< 0.0001*
Elevation of Cr (*n*, %)	38 (50)	14 (2.8)	< 0.001*
C3 (g/L)	1.018 ± 0.322	0.948 ± 0.330	0.725
C4 (g/L)	0.217 ± 0.112	0.173 ± 0.102	0.004*

M (Q1–Q3) represented median (quartile 1to quartile 3)

*Statistical significance

### Comparison of IgG4-RD patients with/without PAO/PA

Compared with non-PAO/PA patients, PAO/PA patients were older at disease onset, had a higher male/female ratio, but had shorter disease duration and lower percentage of an allergic history (*P* < 0.001, *P* < 0.001, *P* < 0.001, and *P* < 0.001, respectively; Table 1). The number of organs involved and IgG4-RD RI were comparable in patients with/without PAO/PA. However, patients with PAO/PA had a higher percentage of single organ involvement than those without PAO/PA (*P* < 0.001).

Patients with PAO/PA had a higher percentage of back pain, abdominal pain, and lower limb edema than those with PAO/PA (4.2% [*P* < 0.001], 15.9% [*P* = 0.005], and 1.6% [*P* < 0.001], respectively). In contrast, patients with PAO/PA had a lower percentage of submandibular gland enlargement, lacrimal gland enlargement, parotid gland enlargement, and nasal congestion than those without PAO/PA (42.8% [*P* < 0.001], 45.6% [*P* < 0.001], 13.3% [*P* = 0.042], and 21.3% [*P* < 0.001], respectively) (Table 2). Consistent with onset symptoms, patients with PAO/PA had a lower percentage of submandibular gland, lacrimal gland, and paranasal involvement (all *P* < 0.001) (Table 2).

Compared to patients with PAO/PA, those with PAO/PA had higher levels of ESR, hsCRP, and IgA (*P* < 0.0001, *P* < 0.0001, and *P* = 0.02, respectively) (Table 3). However, patients with IgG4-related PAO/PA had lower levels of serum IgG4 and IgE than those without IgG4-related PAO/PA (8310 [3250, 17,075] mg/L, 332 [119, 720.5] KU/L; *P* < 0.0001 and *P* = 0.025, respectively) (Table 3). Additionally, serum IgG4 levels were higher in patients with PAO/PA and other organs affected (5270 [2395, 11,910] mg/L) than in those with only PAO/PA (2418 [1583, 4638] mg/L) (*P* = 0.001).

### CD19^+^CD24^−^CD38^hi^ plasmablast/plasma cells

Overall, 93 patients showed CD19^+^CD24^−^CD38^hi^ plasmablast/plasma cells at baseline. No statistical significant difference in CD19^+^CD24^−^CD38^hi^ plasmablast/plasma cell was found between PAO/PA and non-PAO/PA patients. However, the percentage of CD19^+^CD24^−^CD38^hi^ plasmablast/plasma cells was lower in patients with only PAO/PA (2.22% [Q1–Q3, 1.87–5.90%]) than in those with PAO/PA and other organ involvement (8.5% [Q1–Q3, 4.52–16.90%]) or non-PAO/PA (5.58% [Q1–Q3, 2.99–10.50%]) (*P* = 0.015 and *P* = 0.023, respectively).

### Vessels distribution of IgG4-related PAO/PA

**Fig. 2:**
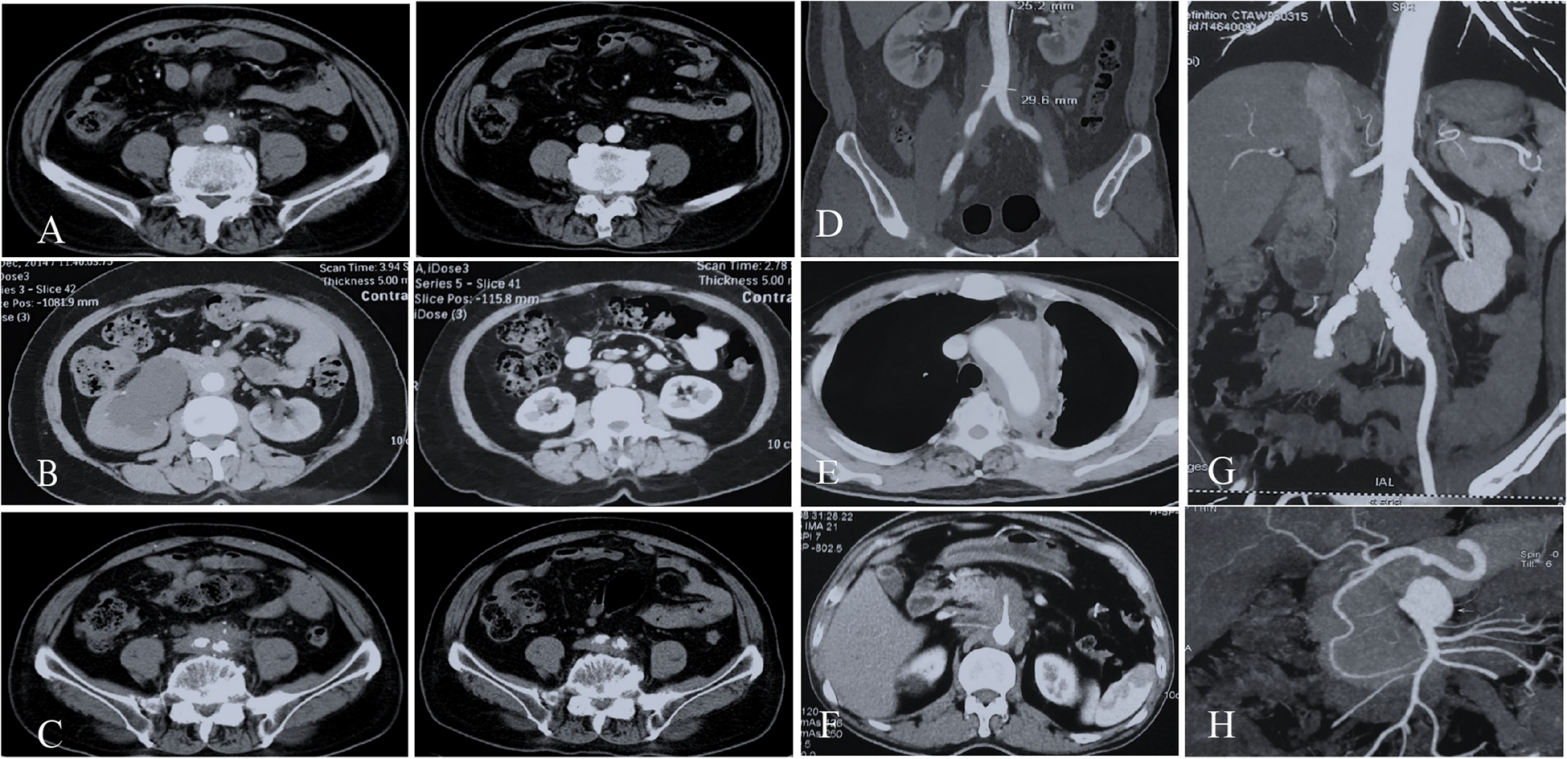
Characteristic imaging findings of immunoglobulin G4-related disease aortitis/periaortitis and periarteritis. **a** A 69-year-old man with periaortitis before and after treatment. **b** A 52-year-old woman with periaortitis and hydronephrosis before and after treatment. **c** The affected iliac artery before and after treatment. **d**, **e** A 54-year-old man with thoracic aorta, abdominal, and iliac artery involvement. **f** Superior mesenteric artery involvement in a 64-year-old man. **g**, **h** Aneurysmal dilation of the abdominal aorta and superior mesenteric artery

Characteristic imaging findings IgG4-related PAO/PA are shown in Fig. 2. Of 89 IgG4-RD patients with PAO/PA, the abdominal aorta was the most affected vessel (74, 83.1%), followed by the iliac artery (63, 70.8%), thoracic aorta (12, 13.5%), and other vessels, including the superior mesenteric artery (6, 6.7%), renal artery (6, 6.7%), common carotid artery (3, 3.4%), and subclavian artery (2, 2.2%), revealing vascular stenosis in 3 patients. Beside soft tissue around the vessels, 27 patients had calcification of the aortic wall, 22.5% had diffuse thickening of the abdominal aortic wall, and 10.1% had aneurysmal dilation of the aorta (Table 4). Abdominal aorta and iliac artery lesions were more common in male patients than in female patients (*P* = 0.024 and *P* = 0.035, respectively; Table 4). However, the ratio of patients with thoracic aorta and other large vessels affected was higher in female patients than in male patients (*P* = 0.048 and *P* = 0.004, respectively; Table 4). Moreover, female patients had a lower percentage of aortic wall calcification than male patients (*P* = 0.047; Table 4).

**Table 4:** Vascular distribution of IgG4-RD with PAO/PA

Vessels affected (***n***, %)	Total (***n*** = 89)	Male (***n*** = 76)	Female (***n*** = 13)	***P*** value
Abdominal aorta	74 (83.1)	66 (86.8)	8 (61.5)	0.024*
Iliac artery	63 (70.8)	57 (75.0)	6 (46.2)	0.035*
Thoracic aorta	12 (13.5)	8 (10.5)	4 (30.8)	0.048*
Other vessels	12 (13.5)	7 (9.2)	5 (38.5)	0.004*
Calcification of vessel wall	27 (30.3)	26 (34.2)	1 (7.7)	0.047*
Diffuse thickening of the abdominal aortic wall	20 (22.5)	16 (21.1)	4 (30.8)	0.438
Aneurysm	9 (10.1)	9 (11.8)	0 (0.0)	0.346
Type 1	5 (5.6)	2 (2.6)	3 (23.1)	0.003*
Type 2	74 (83.1)	66 (86.8)	8 (61.5)	0.024*
Type 2a	15 (16.9)	13 (17.1)	2 (15.4)	1.000
Type 2b	52 (58.4)	47 (83.9)	5 (38.5)	0.114
Type 2c	7 (7.9)	6 (7.9)	1 (7.7)	1.000
Type 3	7 (7.9)	6 (7.9)	1 (7.7)	0.980
Type 4	3 (3.4)	2 (2.6)	1 (7.7)	0.381

*Statistical significance

According to the distribution of IgG4-related PAO/PA, type 2, involvement of abdominal aorta and iliac artery, was the most prevalent (74, 83.1%), especially type 2b (52, 58.4%), followed by type 2a (15, 16.9%), type 2c (7, 7.9%), type 3 (7, 7.9%), type 1 (5, 5.6%), and type 4 (3, 3.4%) (Table 4). Additionally, no statistical significance in vessel distribution was found between patients with PAO/PA alone and those with PAO/PA and other organ involvement.

Further, 55 (61.8%) patients with PAO/PA had hydronephrosis, including 47 (61.8%) male patients and 8 (61.5%) female patients. Forty-three (48.3%) patients developed impairment of renal function caused by ureteral obstruction. Of patients with severe obstruction, double J (D-J) stent drainage was the first option to relieve obstruction, and 31 (34.8%) PAO/PA patients had D-J stent drainage. With regard to other organs affected, the rates of lymph node, pancreas, submandibular gland, lung, prostate, bile duct, lacrimal gland, parotid gland, paranasal sinus, and thyroid gland involvement were 37.1%, 29.2%, 27.0%, 16.9%, 15.8%, 12.4%, 12.4%, 10.1%, 9.0%, and 2.2%, respectively (Table 2).

### Clinical features among the four types of vessel involvement groups

We compared the clinical characteristics at baseline of IgG4-related PAO/PA patients with different vessel distributions (type 1, type 2a, type 2b, type 2c, type 3, and type 4) in supplementary table 1. The results indicated that male patients were predominant in type 2 than in type 1 (*P* = 0.018). Among all the groups, IgG4-RD RI was highest in type 2a (*P* = 0.047). The percentage of creatinine elevation caused by ureter obstruction was much higher in type 2 and type 3 than in the others. There was no statistical significance in the other clinical features among the four groups.

### Treatment efficacy in patients with PAO/PA

Patients with PAO/PA were treated with glucocorticoids (GCs) or GCs combined with immunosuppressant agents (GCs plus immunosuppressant agent).

Except for 18 (20.2%) patients who received GC monotherapy, the other patients were treated with GCs plus cyclophosphamide (CYC) (*n* = 52, 58.4%), GCs plus mycophenolate mofetil (MMF) (*n* = 18, 20.2%), and GCs plus leflunomide (*n* = 1, 1.1%). Forty-one (46.1%) patients received combined treatment with tamoxifen.

After 6 months of treatment, 34 (38.2%) patients achieved reduction of perivascular soft tissues > 70%, 39 (43.8%) achieved reduction between 31 and 70%, and 16 (18.0%) had reduction < 30%. Compared with male patients with PAO/PA, a higher percentage of female patients had reduction of perivascular soft tissues < 30% (*P* = 0.01). In 31 (34.8%) patients who had D-J stent drainage, 22 (71.0%) patients had successful stent extubation, and the median time of extubation was 6 (3–13.5) months. Of 43 patients with renal function impairment, 72.1% patients with renal insufficiency at baseline showed normalization of the serum creatinine during the follow-up. Twelve (27.9%) patients’ serum creatinine level decreased but remained above the normal range. The eGFR before treatment was 46.0 ± 18.0 ml/min/1.73 m^2^, and it increased to 66.7 ± 18.8 ml/min/1.73 m^2^ after therapy (supplementary figure 1).

Nine (10.1%) patients who had aneurysmal dilatation at baseline received initial dose of GCs (0.5 mg/kg) combined with immunosuppressant agent. We monitored changes of the dilated vessels by CT scan every 3~6 months in the first year after diagnosis. After treatment, there was no significant change in the dilatation scope and dilated diameter of PAO/PA. No patient experienced aneurysmal rupture.

The IgG4-RD RI and ESR, hsCRP, serum IgG4, and IgE levels reduced significantly after treatment. The serum IgG4 level returned to the normal range in 57.3% of patients with PAO/PA, and the serum IgE level returned to the normal range in 33.7% of patients after 6 months of treatment.

Five (5.6%) patients relapsed during the follow-up with a median recurrence time of 21 (15.5–33) months; all of them had two or multiple organs involved at baseline and relapsed in other organs beyond the blood vessels. No significant difference in the relapse rate was found between male and female patients.

### Comparison between IgG4-related PAO/PA patients with shrinkage of soft tissues around vessels > 70% and < 30%

In order to clarify the differences of IgG4-related PAO/PA patients who had significantly different treatment efficacy, we compared the clinical features between the more than 70% responder group (group A) and less than 30% responder group (group B) (supplementary table 2). Patients in group A were older and more predominantly male than those in group B. Regarding organ involvement, the percentage of lymphadenopathy was higher in group A than in group B (*P* = 0.01), while other organs involvement was comparable between group A and group B. The IgG3 level was higher in group A than in group B (*P* = 0.006). There was no statistical significance in other clinical features between the two groups.

## Discussion

To our knowledge, this is the largest prospective cohort of IgG4-RD patients with PAO/PA who were compared to patients without PAO/PA. We compared the clinical manifestations in IgG4-RD with or without PAO/PA in a large prospective cohort from China. In addition, the treatment efficacy in PAO/PA patients was evaluated.

IgG4-related PAO/PA was associated with male predominance and could affect multiple organs [10–12], and it affects 60–85% of male patients [10, 13, 23]; thus, it is different from Takayasu arthritis (TA) with a young female predominance. Male PAO/PA patients were older at disease onset than female patients. Consistent with other reported IgG4-related PAO/PA, gender has a strong influence on the pattern of vascular involvement and consequently on clinical presentation; women have a higher involvement of supradiaphragmatic vessels, whereas men have abdominal vessel involvement [9, 13, 24].

According to allergy, a substantial percentage of IgG4-RD patients had histories of allergy [8], the percentage of allergy was higher in patients with IgG4-related dacryoadenitis/sialadenitis (DS) than in those with non-IgG4 DS [7], and non-PAO/PA patients had a higher percentage of allergy than PAO/PA patients [5]. Our result also showed that allergic diseases were significantly more common in patients with lacrimal and/or salivary gland lesions than those without, indicating that allergies are related to different clinical phenotypes. The demographic features of our PAO/PA patients were consistent with those in other studies [5, 25, 26]. The shorter disease duration indicated that the severe disease activity needs urgent treatment, such as severe constitutional symptoms, back pain, higher inflammatory parameters, or renal function impairment due to ureter obstruction. Whether the low rate of allergy history of PAO/PA patients compared with those without suggested different triggering factors or etiology remained to be further elucidated.

The onset symptoms of PAO/PA patients were non-specific, including pain (often back or abdominal pain, as chest or groin pain was relatively rare seen), edema of the lower limbs, and dyspnea [27]. In our cohort, the most common onset symptoms were back pain and abdominal pain, followed by lymph node swelling, submandibular gland enlargement, and lower limb edema in most patients with multiorgan involvement. However, back pain or abdominal pain is an atypical symptom, so abdominal CT or MRI must be conducted in patients with such symptoms for early detection of PAO/PA. Of PAO/PA patients, the most prevalent type of vessel distribution was type 2, consistent with Ozawa et al.’s study [5, 26]. However, PAO/PA patients had a much lower rate of DS and paranasal sinusitis than non-PAO/PA patients, which is consistent with our previous study finding that patients without DS had a higher percentage of aorta or larger blood vessel involvement [7]. Nearly half IgG4-related PAO/PA patients in our cohort developed renal function impairment due to obstruction of the ureter. The percentage of creatinine elevation was higher in type 2, especially in type 2b, indicating that urgent treatment (surgical intervention if necessary) may prevent those patients from experiencing deteriorating renal function.

Elevation of ESR and hsCRP was an indicator of vascular wall inflammation in large-vessel vasculitis, such as chronic periaortitis and TA [5, 26, 28, 29]. It has been reported that higher level of CRP is a hallmark for detecting perivascular involvement in IgG4-RD [25, 28]. The mechanism of CRP elevation in IgG4-related PAO/PA needs to be further elucidated. Our data demonstrated that PAO/PA patients had a higher white blood cell count, ESR, and hsCRP, but lower blood hemoglobulin, serum IgG4, and IgE levels than IgG4-RD patients without PAO/PA. Besides, in patients with only PAO/PA involvement, serum IgG4 levels were also lower than those in patients with PAO/PA and other organ involvement [7]. In our cohort, more than 70% of PAO/PA patients had elevated ESR and hsCRP, which is much higher than that reported by Mizushima et al., in which only a small proportion of patients had elevated hsCRP [26]. Patients with only PAO/PA involvement also had low levels of CD19^+^CD24^−^CD38^hi^ plasmablast/plasma cells compared with patients with PAO/PA and other organ involvement or non-PAO/PA patients. Circulatory inflammation was prominent, but low serum IgG4 in PAO/PA patients compared with non-PAO/PA patients, indicating different disease pathogenesis that needs to be elucidated. Dacryoadenitis, higher serum IgG4, T-IgE, and higher circulating plasmablasts were risk factors for disease relapse [30–33], and the above parameter was lower in PAO/PA patients, which may indicate a lower relapse rate.

IgG4-RD with PAO/PA might be a distinct spectrum of IgG4-RD, as it is characterized by prominent fibrosis, sparse lymphoplasmacytic infiltration, fewer extra-nodal germinal centers, and mildly elevated serum IgG1, IgG4, and IgE concentrations [5, 28, 34]. Recently, a pioneer approach to classifying IgG4-RD into proliferative subtype and fibrotic subtype was reported [35]. The characteristics of the fibrotic subtype usually involve extra-glandular sites and can involve a body region rather than a specific organ, including retroperitoneum fibrosis, sclerosing mesenteritis, and fibrosing mediastinitis. By comparison of IgG4-RD without PAO/PA in clinical manifestations, organ involvement, and laboratory parameters, IgG4-related PAO/PA patients showed distinct characteristics that could be classified into the fibrotic subtype.

Aortitis needs to be treated urgently because inflammatory aortic aneurysms may have a large diameter or high enlargement rate and are at a high risk of rupture. Patients with chronic periaortitis were often treated with medium to high dose GCs [14, 27]. Immunosuppressants are steroid-sparing treatments for PAO/PA patients. Previous studies indicated that CYC and MMF and rituximab were effective as induction therapy [10, 22, 36, 37]. For patients with ureter obstruction, quick relief from the obstruction by intra-ureteral stenting with a D-J stent could prevent further kidney damage. If obstruction is absent or mild and there is no renal function impairment, an immunosuppressive regimen is the first option. When moderate to severe ureteral obstruction and/or renal impairment are present, ureteral drainage must be the priority, followed by immunosuppressive therapy [38]. In view of the report of ruptured aneurysms after GC therapy in the literature [39], we used the strategy of moderate dose GC (0.5 mg/kg) combination and immunosuppressive therapy for nine patients with aneurysmal dilatation. There was no significant change in the scope and diameter of dilatation after treatment, and no patient experienced aneurysmal rupture. In our cohort, most of the PAO/PA patients were treated with GCs combined with immunosuppressant, and more than 90% of patients achieved CR. More female PAO/PA patients could not achieve reduction of perivascular soft tissues for > 30%, suggesting that female patients tended to be more resistant to treatment than male patients. In addition, 71.0% of our patients with ureteral obstruction were successfully extubated with a median time of 6 months. The reduction of ESR, hsCRP, serum IgG4, and T-IgE levels was also an indicator of treatment efficacy. Compared with IgG4-DS patients, a higher proportion of PAO/PA patients managed to achieve normal serum IgG4 and IgE levels after treatment [7].

This study had some limitations. First, this is a single-center study. Second, the follow-up time was relatively short. Third, PET-CT is a more sensitive imaging test in evaluating vascular lesions; however, due to its high cost, most of the patients did not undertake PET-CT. Fourth, a more comprehensive investigation of pathogenesis needs to be conducted.

## Conclusions

Our study indicates that IgG4-related PAO/PA is distinct from non-PAO/PA in demographic features, organ involvement distribution, inflammatory markers, and serum IgG4 and IgE level. The most common affected vessel was the abdominal aorta, and most patients responded well with treatment. As IgG4-related PAO/PA is a spectrum of the fibrosis subtype of IgG4-RD, disease relapse is less likely to occur in patients with IgG4-related PAO/PA than in those without PAO/PA. Our study’s findings could promote the understanding of IgG4-related PAO/PA in clinical characteristics and treatment efficacy.

## Supplementary information

**Additional file 1: Supplementary Figure 1.** Improvement of eGFR of IgG4 -related PAO/PA with renal impairment after treatment (*n* = 43). eGFR, estimated glomerular filtration rate.

**Additional file 2: Supplementary Table 1.** Comparison of characteristics at baseline among four types of vessel involvement groups. Q1, quartile 1; Q3, quartile 3; SD, standard deviation; WBC, white blood cell; HgB, hemoglobin; PLT, platelet; EOS, eosinophils; ESR, estimated sedimentation rate; hsCRP, hypersensitive C-reactive protein; Ig, immunoglobulin; C3, complement 3; C4, complement 4; Cr, creatinine; IgG4-RD RI, immunoglobulin G4-related disease responder index.

**Additional file 3: Supplementary Table 2.** Comparison of characteristics at baseline between the more than 70% responder group (Group A) and less than 30% responder group (Group B). Q1, quartile 1; Q3, quartile 3; SD, standard deviation; WBC, white blood cell; HgB, hemoglobin; PLT, platelet; EOS, eosinophils; ESR, estimated sedimentation rate; hsCRP, hypersensitive C-reactive protein; Ig, immunoglobulin; C3, complement 3; C4, complement 4; Cr, creatinine; IgG4-RD RI, immunoglobulin G4-related disease responder index; T-Ig, total immunoglobulin.

## Data Availability

The dataset analyzed in this paper is available from the corresponding author on reasonable request, and with appropriate additional ethical approvals, where necessary.

## Abbreviations

Ig: Immunoglobulin
IgG4-RD: Immunoglobulin G4-related disease
PAO/PA: Aortitis/periaortitis and periarteritis
CT: Computed tomography
MRI: Magnetic resonance imaging
PET-CT: Positron emission tomography/computed tomography
IgG4-RD RI: IgG4-RD responder index
D-J stent: Double J stent
ESR: Erythrocyte sedimentation rate
hsCRP: Hypersensitive C-reactive protein
IgG: Immunoglobulin G
GCs: Glucocorticoids
IM: Immunosuppressant
CYC: Cyclophosphamide
MMF: Mycophenolate mofetil
eGFR: Estimated glomerular filtration rate
TA: Takayasu arthritis
IgG4-DS: IgG4-related dacryoadenitis/sialadenitis
C3: Complement 3
C4: Complement 4
