# Supplementary material for: IgG4-related aortitis/periaortitis and periarteritis: a distinct spectrum of IgG4-related disease
Source: Arthritis Res Ther. 2020 May 4;22:103. doi: 10.1186/s13075-020-02197-w (PMC7197178; doi:10.1186/s13075-020-02197-w)
Supplement: Supplementary file 2 — Additional file 2: Supplementary Table 1. Comparison of characteristics at baseline among four types of vessel involvement groups. Q1, quartile 1; Q3, quartile 3; SD, standard deviation; WBC, white blood cell; HgB, hemoglobin; PLT, platelet; EOS, eosinophils; ESR, estimated sedimentation rate; hsCRP, hypersensitive C-reactive protein; Ig, immunoglobulin; C3, complement 3; C4, complement 4; Cr, creatinine; IgG4-RD RI, immunoglobulin G4-related disease responder index. [file 13075_2020_2197_MOESM2_ESM.docx]

| **Characteristics at baseline** | **Type1 (n=5)** | **Type2** | | | | | **Type 3**  **(n=7)** | **Type 4**  **(n=4)** | ***P-*value** |
| --- | --- | --- | --- | --- | --- | --- | --- | --- | --- |
|  |  | **Type2a (n=15)** | | **Type2b (n=52)** | | **Type 2c**  **(n=7)** |  |  |  |
| **Demographic features** |  |  |  | |  | |  |  |  |
| Age | 56.4±11.1 | 61.2±7.4 | 58.7±11.3 | | 58.1±8.9 | | 54.7±11.6 | 49.7±26.6 | 0.768 |
| Male/female ratio | 0.67:1 | 6.5:1 | 9.4:1 | | 6:1 | | 6:1 | 2:1 | 0.07 |
| Disease duration(months) M(Q1-Q3) | 36.0 (2-120) | 12.0 (3.0-36.0) | 6.0 (2.0-34.5) | | 5.0 (4.0-15.0) | | 24.0 (4.0-60.0) | 2.3±1.5 | 0.257 |
| History of allergy (n, %) | 2 (40) | 6 (40) | 12 (23.1) | | 2 (28.6) | | 3 (42.9) | 0 (0) | 0.552 |
| **Organs affected(n,%)** |  |  |  | |  | |  |  |  |
| Pancreas | 0 (0.0) | 8 (53.3) | 15 (28.8) | | 2 (28.6) | | 0 (0.0) | 1 (33.3) | 0.101 |
| Bile duct | 0 (0.0) | 3 (20.0) | 6 (11.5) | | 1 (14.3) | | 1 (14.3) | 0 (0.0) | 0.847 |
| Submandibular gland | 2 (40.0) | 8 (53.3) | 11 (21.2) | | 2 (28.6) | | 1 (14.3) | 0 (0.0) | 0.140 |
| Lacrimal gland | 2 (40.0) | 4 (26.7) | 4 (7.7) | | 1 (14.3) | | 0 (0.0) | 0 (0.0) | 0.116 |
| Parotid gland | 0 (0.0) | 4 (26.7) | 4 (7.7) | | 1 (14.3) | | 0 (0.0) | 0 (0.0) | 0.246 |
| Lung | 1 (20.0) | 5 (33.3) | 8 (15.4) | | 0 (0.0) | | 0 (0.0) | 1 (33.3) | 0.266 |
| Kidney | 0 (0.0) | 2 (13.3) | 2 (3.8) | | 1 (14.3) | | 0 (0.0) | 1 (33.3) | 0.567 |
| Prostate | 0 (0.0) | 3 (23.1) | 7 (14.9) | | 2 (33.3) | | 0 (0.0) | 0 (0.0) | 0.577 |
| Lymph node | 2 (40.0) | 6 (40.0) | 19 (36.5) | | 2 (28.6) | | 4 (57.1) | 0 (0.0) | 0.658 |
| Paranasal Sinus | 1 (20.0) | 1 (6.7) | 5 (9.6) | | 0 (0.0) | | 1 (14.3) | 0 (0.0) | 0.836 |
| Thyroid gland | 0 (0.0) | 0 (0.0) | 2 (3.8) | | 0 (0.0) | | 0 (0.0) | 0 (0.0) | - |
| Pituitary gland | 0 (0.0) | 0 (0.0) | 2 (3.8) | | 0 (0.0) | | 0 (0.0) | 0 (0.0) | - |
| Skin | 0 (0.0) | 0 (0.0) | 1 (1.9) | | 0 (0.0) | | 0 (0.0) | 0 (0.0) | - |
| Number of organs affected(mean±SD) | 2.8±2.2 | 4.0±2.3 | 2.7±1.7 | | 2.7±2.3 | | 2.0±1.0 | 2.3±0.6 | 0.294 |
| **Laboratory parameters** |  |  |  | |  | |  |  |  |
| WBC (10^9^/L) | 9.89±2.70 | 7.86±3.11 | 7.97±2.98 | | 6.21±1.37 | | 6.85±1.36 | 9.77±1.12 | 0.01* |
| HGB (g/L) | 147±16 | 126±24 | 125±19 | | 122±24 | | 140±24 | 123±12 | 0.07 |
| PLT (10^9^/L) | 288±111 | 210±100 | 240±71 | | 225±64 | | 251±86 | 321±221 | 0.242 |
| EOS% | 4.6 (1.1-11.2) | 2.5 (1.0-5.1) | 2.6 (1.0-4.5) | | 2.8 (0.3-5.2) | | 2.1 (0.8-5.5) | 2.1±3.1 | 0.897 |
| ESR (mm/h) | 39 (11-71) | 62 (25-81) | 49 (22-79) | | 42 (11-73) | | 37 (2-67) | 28±24 | 0.756 |
| hsCRP (mg/L) | 2.52 (1.59-18.45) | 3.3 (1.72-35.26) | 8.93 (3.80-24.45) | | 6.92 (4.30-30.83) | | 3.84 (1.78-32.71) | - | 0.535 |
| IgG (g/L) | 17.58±13.89 | 20.41±7.90 | 19.29±6.50 | | 25.00±15.09 | | 20.93±9.76 | 15.80±7.35 | 0.455 |
| IgA (g/L) | 2.63±1.28 | 2.49±1.32 | 2.44±1.02 | | 2.72±1.29 | | 2.87±1.55 | - | 0.910 |
| IgM (g/L) | 0.72±0.46 | 0.76±0.36 | 0.97±0.65 | | 0.95±0.21 | | 1.12±0.54 | - | 0.303 |
| IgG1 (mg/L) | 6800 (6135-9080) | 9645 (7893-13450) | 9360 (7980-11100) | | 9690 (8600-12500) | | 12400 (7110-18850) | 7660±1855 | 0.130 |
| IgG2 (mg/L) | 3560 (2410-6385) | 5495 (4350-10825) | 6220 (4080-7255) | | 7250 (5450-9030) | | 4605 (2665-5460) | 4497±2566 | 0.072 |
| IgG3 (mg/L) | 205 (102-519) | 948 (382-1253) | 506 (231-839) | | 247 (216-1420) | | 270 (202-914) | 283±171 | 0.056 |
| IgG4 (mg/L) | 1060 (678-26285) | 5130 (2240-20400) | 4070 (2383-7253) | | 3870 (1680-17400) | | 4600 (475-5270) | 3485±4087 | 0.608 |
| IgE (KU/L) | 72.6 (43.8-612.5) | 129.0 (78.2-184.0) | 271.5 (123.5-725.5) | | 214.5 (50.4-431.3) | | 73.5 (20.0-274.0) | - | 0.098 |
| C3 (g/L) | 1.157±0.257 | 0.890±0.298 | 1.057±0.321 | | 1.079±0.321 | | 0.855±0.416 | 0.985±0.283 | 0.708 |
| C4 (g/L) | 0.294±0.155 | 0.208±0.144 | 0.224±0.987 | | 0.257±0.122 | | 0.131±0.078 | 0.166±0.072 | 0.701 |
| Cr elevation (n,%) | 0 (0.0) | 3 (20.0) | 34 (65.4) | | 4 (57.1) | | 2 (28.6) | 0 (0.0) | 0.005* |
| **Disease activity** |  |  |  | |  | |  |  |  |
| IgG4-RD RI | 9.0±7.0 | 13.7±5.7 | 10.7±5.3 | | 11.1±6.9 | | 6.9±2.3 | 7.0±1.7 | 0.047* |

Supplementary table 1. Comparison of characteristics at baseline among four types of vessel involvement groups.

Q1, quartile 1; Q3, quartile 3; SD, standard deviation; WBC, white blood cell; HgB, hemoglobin; PLT, platelet; EOS, eosinophils; ESR, estimated sedimentation rate; hsCRP, hypersensitive C-reactive protein; Ig, immunoglobulin; C3, complement 3; C4, complement 4; Cr, creatinine; IgG4-RD RI, immunoglobulin G4-related disease responder index
