# Supplementary material for: IgG4-related aortitis/periaortitis and periarteritis: a distinct spectrum of IgG4-related disease
Source: Arthritis Res Ther. 2020 May 4;22:103. doi: 10.1186/s13075-020-02197-w (PMC7197178; doi:10.1186/s13075-020-02197-w)
Supplement: Supplementary file 3 — Additional file 3: Supplementary Table 2. Comparison of characteristics at baseline between the more than 70% responder group (Group A) and less than 30% responder group (Group B). Q1, quartile 1; Q3, quartile 3; SD, standard deviation; WBC, white blood cell; HgB, hemoglobin; PLT, platelet; EOS, eosinophils; ESR, estimated sedimentation rate; hsCRP, hypersensitive C-reactive protein; Ig, immunoglobulin; C3, complement 3; C4, complement 4; Cr, creatinine; IgG4-RD RI, immunoglobulin G4-related disease responder index; T-Ig, total immunoglobulin. [file 13075_2020_2197_MOESM3_ESM.docx]

| **Characteristics at baseline** | **Group A(n=34)** | **Group B(n=16)** | ***P* -value** |
| --- | --- | --- | --- |
| **Demographic features** |  |  |  |
| Age (years) | 60.3±9.4 | 54.3±7.8 | 0.03* |
| Male/Female ratio | 16/1 | 1.67/1 | 0.004* |
| Disease duration (month),  M (Q_1_-Q_3_) | 8 (3-36) | 14 (5-57) | 0.257 |
| History of allergy (n, %) | 9 (26.5) | 4 (25.0) | 0.912 |
| **Vessel distribution(n,%)** |  |  |  |
| Type 1 | 1(2.9) | 3(18.8) | 0.055 |
| Type 2a | 6(17.6) | 2(12.5) | 0.643 |
| Type 2b | 21 (61.8) | 8 (50.0) | 0.432 |
| Type 2c | 4 (11.8) | 2 (12.5) | 0.941 |
| Type 3 | 2(5.9) | 1(6.3) | 0.322 |
| Type 4 | 0(0.0) | 0(0.0) | - |
| **Symptoms at disease onset (n, %)** |  |  |  |
| Back pain | 14 (41.2) | 7 (43.8) | 0.863 |
| Lymph node swelling | 11 (32.4) | 2(12.5) | 0.135 |
| Abdominal pain | 14 (41.2) | 3 (18.8) | 0.118 |
| Submandibular gland enlargement | 4 (11.8) | 4 (25.0) | 0.234 |
| Lacrimal gland enlargement | 3 (8.8) | 2 (12.5) | 0.686 |
| Lower limb edema | 8(23.5) | 2 (12.5) | 0.363 |
| Cough | 6 (17.6) | 4 (25.0) | 0.544 |
| Nausea and vomiting | 6 (17.6) | 2 (12.5) | 0.643 |
| Jaundice | 3 (8.8) | 1(6.3) | 0.754 |
| Parotid gland enlargement | 2 (5.9) | 1 (6.3) | 0.959 |
| Nasal congestion | 1 (2.9) | 2 (12.5) | 0.237 |
| Itching | 3(8.8) | 1 (6.3) | 0.754 |
| **Organ affected (n, %)** |  |  |  |
| Lymph node | 16 (47.1) | 2 (12.5) | 0.018* |
| Submandibular gland | 7 (20.6) | 4 (25.0) | 0.725 |
| Pancreas | 13 (38.2) | 2 (12.5) | 0.178 |
| Lung | 7 (20.6) | 2 (12.5) | 0.487 |
| Lacrimal gland | 3 (8.8) | 3 (18.8) | 0.314 |
| Parotid gland | 2 (5.9) | 2 (12.5) | 0.421 |
| Bile duct | 5 (14.7) | 3 (18.8) | 0.716 |
| Paranasal sinus | 2 (5.9) | 3 (18.8) | 0.157 |
| Prostate | 7 (21.9) | 1 (10.0) | 0.197 |
| Kidney | 3 (8.8) | 1 (6.3) | 1.000 |
| Thyroid | 0 (0.0) | 1 (6.3) | - |
| Pituitary | 0 (0.0) | 2 (12.5) | - |
| Skin | 1 (2.9) | 0 (0.0) | - |
| Number of organs involved (mean±SD) | 3.0±1.9 | 2.8±2.0 | 0.397 |
| Number of single organ involvement(n,%) | 8(23.5) | 7(43.8) | 0.191 |
| **Laboratory parameters** |  |  |  |
| HGB (g/L) | 125±23 | 129±24 | 0.531 |
| WBC (10^9^/L) | 7.9±2.9 | 8.5±2.6 | 0.501 |
| PLT (10^9^/L) | 248±91 | 266±67 | 0.500 |
| EOS(%) | 2.3(0.7-3.9) | 2.3(0.7-4.7) | 0.809 |
| ESR(mm/h), M (Q_1_-Q_3_) | 53 (22-82) | 36 (8-73) | 0.248 |
| hsCRP(mg/L), M (Q_1_-Q_3_) | 9.06 (4.3-26.9) | 3.99 (2.0-30.83) | 0.266 |
| IgG (g/L) | 20.93±7.60 | 17.68±7.45 | 0.175 |
| IgA (g/L) | 2.53±1.10 | 2.90±1.24 | 0.324 |
| IgM(g/L) | 0.92±0.35 | 0.78±0.31 | 0.226 |
| IgG1 (mg/L), M (Q_1_-Q_3_) | 9210 (7995-10025) | 9025 (7095-12350) | 0.967 |
| IgG2 (mg/L), M (Q_1_-Q_3_) | 5630 (3905-8280) | 5480 (3560-6220) | 0.456 |
| IgG3 (mg/L), M (Q_1_-Q_3_) | 443 (269-1090) | 228 (138-332) | 0.006* |
| IgG4 (mg/L), M (Q_1_-Q_3_) | 3460(2285-6923) | 2678 (1485-8693) | 0.454 |
| T-IgE (KU/L), M (Q1-Q3) | 184(110.5-560.5) | 262.5 (61.4-335.5) | 0.483 |
| Elevation of Cr (n, %) | 21 (61.8) | 8 (50.0) | 0.432 |
| C3 (g/L) | 1.077±0.335 | 1.099±0.239 | 0.845 |
| C4 (g/L) | 0.222±0.117 | 0.278±0.082 | 0.158 |
| **Disease activity** |  |  |  |
| Baseline IgG4-RD RI | 11.6±5.5 | 10.3±6.0 | 0.322 |

Supplementary table 2. Comparison of characteristics at baseline between the more than 70% responder group (Group A) and less than 30% responder group (Group B).

Q1, quartile 1; Q3, quartile 3; SD, standard deviation; WBC, white blood cell; HgB, hemoglobin; PLT, platelet; EOS, eosinophils; ESR, estimated sedimentation rate; hsCRP, hypersensitive C-reactive protein; Ig, immunoglobulin; C3, complement 3; C4, complement 4; Cr, creatinine; IgG4-RD RI, immunoglobulin G4-related disease responder index; T-Ig, total immunoglobulin
